# Perceived parental relationship quality and adolescent conscious bias: important links between affect, agency, gender, and race/ethnicity

**DOI:** 10.3389/fpsyg.2025.1644117

**Published:** 2025-11-27

**Authors:** Amanda Terrell, Michael J. Merten, Thulitha Wickrama

**Affiliations:** 1 School of Human Environmental Sciences, University of Arkansas-Fayetteville, Fayetteville, AR, United States; 2 Department of Child, Youth and Family Studies, University of Nebraska-Lincoln, Lincoln, NE, United States

**Keywords:** adolescents, parenting, attitudes, prejudice, minority, human agency, bias

## Abstract

**Introduction:**

Guided by attachment, social cognitive, and ecological systems theories, this study examined the direct and indirect effects of perceived parent-adolescent relationship quality on youths' conscious bias toward individuals facing socioeconomic or minority-related challenges.

**Methods:**

Data were collected from 702 U.S. 18-year-olds via Qualtrics online panels. Measures included parent relationship quality, negative affect, human agency, and conscious bias (disregard for minority experiences and blaming attitudes).

**Results:**

The findings revealed that strong parent-teen bonds indirectly influenced conscious bias through psychosocial wellbeing. However, while negative emotions were universally influential, the impact of human agency varied depending on gender and race/ethnicity.

**Discussion:**

Study findings underscore the necessity for a more comprehensive understanding of human agency as personal empowerment, considering gendered socialization practices and cultural nuances across different racial/ethnic groups. Additionally, there is an important need to integrate diversity, equity, and inclusion principles into parental education initiatives.

## Introduction

The concepts of diversity, equity, and inclusion (DEI) represent organizational commitments to recognizing human differences (diversity), ensuring fair treatment and opportunity (equity), and creating environments where all individuals can contribute (inclusion) (Shore et al., 2018). In recent years these concepts have emerged as priorities within educational institutions. Related competencies, including emotional intelligence and empathy, are increasingly recognized as foundational to DEI implementation (Extension Foundation, 2022). One issue that is increasingly targeted by these programs is whether a person's biases are conscious or unconscious. Conscious biases are those (often blaming) attitudes a person is aware of and can articulate (National Center for Cultural Competence, n.d.), such as blaming people for their misfortunes or explicitly disregarding people's discriminatory or marginalized experiences. Efforts to enhance DEI awareness, knowledge, and skills are taking place amidst a contentious backdrop of federal support for social-emotional learning [SEL; Collaborative for Academic, 2022] and mixed state support with several bills proposed to eliminate SEL topics from school curriculum (e.g., Indiana, Oklahoma; Gabriel et al., 2019; Strozewski, 2022). Positive, prosocial youth development is a major emphasis in DEI work, as is preparing adults who work with youth to be “change agents…to create more welcoming and inclusive environments” for young people (National 4-H Council, 2022, para. 1).

Research on the development of bias indicates early emergence of preferences. Infants show own-race preferences by 3 months (Kelly et al., 2005), and toddlers demonstrate in-group favoritism in resource distribution by 3 years (Dunham et al., 2011). Early childhood biases are subsequently shaped through parent-child interactions over time, with parental attitudes predicting children's intergroup attitudes by preschool (Castelli et al., 2008). Early biases become more complex during adolescence through cognitive development and expanded social experiences (Killen and Rutland, 2011). Geraci et al. (2024) found early tendencies for intergroup coalition but late-emerging racial prejudice, suggesting social experiences and parental interactions critically shape racial biases. Similarly, Geraci et al. (2025) demonstrated early emerging gender bias but late-emerging gender prejudice, highlighting how parental socialization differentially impacts bias types across development. Despite these findings, there remains limited focus on families—specifically parents and adolescents—and how parent-child relationships contribute to adolescents' development of biased or prejudicial thinking toward people who are different from them and those who are vulnerable to hardship and marginalization. Despite criticisms of bias and prejudice, the attitude that people should “pull themselves up by their bootstraps” persists—regardless of whether a person or family even has boots Frost and Hoggett, (2008)—and might be reinforced within the family context. Dishion and McMahon (1998) placed parent-teen relationship quality at the center of how parents shape youth attitudes and behaviors toward others. However, when thinking about current DEI issues and initiatives, they are typically discussed and implemented within organizations (such as schools and camps) or addressed with parents of vulnerable populations from an advocacy perspective. There is extremely limited focus on how parents in general are prepared to guide their teenager through DEI issues in the real world—whether their child is of a privileged subgroup experiencing minimal hardship or belonging to a vulnerable population where some individuals overcome risk and others succumb. This is an important research topic, given how parents are primary models of empathic behavior, including through their own social and political behaviors that youth now have widespread access to and often emulate through social media (Zawadzka et al., 2018). Recently, Common Sense Media pointed out that white parents do not typically feel the same pressure to coach their children about *isms*—racism, classism, sexism—as minority parents Filucci, (2021). This issue will continue to grow in importance as the dynamics of racism and classism continue to dominate public discourse and social media engagement.

The present study integrates attachment theory (Bowlby, 1988), social cognitive theory (Bandura, 2001), and ecological systems theory (Bronfenbrenner and Morris, 2006) to examine how parent-adolescent relationships shape conscious bias. Race is conceptualized as a socially constructed category based on perceived physical characteristics, while ethnicity refers to shared cultural heritage, language, and traditions (Helms et al., 2005). These distinctions are critical as they may shape different experiences of discrimination and parental socialization practices. The purpose of the present study was to examine the role of parent-teen relationships in terms of young people's attitudes toward individuals experiencing adversity due to their socioeconomic status or race/ethnicity. Specifically, adolescents' perceived closeness to their parents functions as an indicator of attachment security (Bowlby, 1969), which may be an important link between parental socialization of youth bias (Dunbar et al., 2022; Stern et al., 2022). As an indicator of attachment security, parental relationship quality supports the development of autonomy, agency, and emotion regulation (associated with lower negative affect) and, in turn, may shape cognitive biases (Mikulincer and Shaver, 2022). Of particular interest are youth characteristics, such as gender or belonging to a historically marginalized group, and how those individuals perceive people who are struggling. Individual internalizing characteristics are also important potential links between parent-adolescent bonds and the youth's attitudes toward others.

Parent-child relationships are the first and primary context for shaping how young people view others. From an attachment perspective, this is described as early caregivers heavily contributing to the development of children's internal working models (whether self and others are worthy of love and trustworthy, Joireman et al., 2002). While attachment may develop and function differently for diverse families (Causadias et al., 2022), the ways in which close caregiver-child bonds shape other relationships is widely important. Empirical evidence supports internalizing problems as mediators between parental bonds and social attitudes. Eisenberg et al. (2010) found that emotion regulation, developed through secure attachment, predicts prosocial behavior and empathy. Meta-analytic evidence shows parental warmth reduces internalizing problems, which in turn predict reduced empathic concern (Yan et al., 2019). Studies have further evidenced how dynamics within parent-child relationships, such as problem-solving and conflict management, contribute to the development of empathy in youth (in terms of affect and perspective-taking; Van Lissa et al., (2016). Adolescence is the developmental period when the ability to take on the perspective of another rapidly evolves (Hall et al., 2021). Yet, while parents serve as children's first and arguably most significant relationship in which empathy and related thought processes are (or are not) developed, other internal and behavioral factors also play important roles in adolescents' abilities to understand and empathize with others—particularly others with different characteristics and life circumstances than their own (Silke et al., 2018). Although “parent-child relationship” is a broad term encompassing multiple individual and interpersonal dynamics (e.g., parental knowledge, behaviors, control, and connection with child; Yoo et al., 2013), an adolescent's perception of having a close relationship with their parent can function as a proxy for a wide variety of these dynamics indicating the overall quality of the parent-child relationship (Mak et al., 2020). In fact, some studies suggest that the youth's perspective is paramount in understanding their lived reality (Henry et al., 2008). The present study focuses on how close youth perceive their relationship with their parents to better understand links between these bonds and youth attitudes toward others.

Studies consistently show that poorer parent-child bonds are linked with child and adolescent internalizing problems (Brumariu and Kerns, 2010). Furthermore, there is an inverse relationship between negative (particularly angry or aggressive) affect and empathy that has been widely studied as it relates to peer relationships (e.g., Fung et al., 2015). A person's own mental wellness is an important potential mechanism in the development and practice of social empathy (Vignemont and Singer, 2006), as well as subtle prejudices expressed as blaming attitudes toward people experiencing adversity and misfortune (Önal et al., 2021). In fact, a person's own depressive symptomology, anger, or anxiety can consume their mental focus, thereby limiting their capacity to have concern for or take the perspective of another (Önal et al., 2021). There is also research showing that people experiencing mental health problems have difficulty recognizing emotions in others, which directly impairs empathetic response (Janezic and Arsenault, 2021). Given the link between parent-child relationships and youth internalizing difficulties, the present study examines the role of negative affect as a potential link between parent-child closeness and youth attitudes toward others.

In a nationally-representative, longitudinal study of factors shaping agency and mental health, Williams and Merten (2014) found that better parent-child relationships significantly increased human agency among youth. Agency has been identified as an important contributor to individuals' long-term wellbeing (Williams and Merten, 2014), but is virtually unexplored in terms of if and how it shapes individuals' fundamental attribution errors (or blaming perspectives; Ross, (1977) toward others, particularly based on key contextual factors known to correlate with poorer outcomes. The significance of social schemas and self-projections on others is an important consideration when examining human tendencies to blame people for their misfortunes (Zafari and Koeszegi, 2020). This is evident in the colloquialism, “Pull yourself up by your bootstraps.” The degree to which a person assumes everyone has the capacity, willingness, and ability to rebound from hardship may be significantly linked with their own personal degree of agency. The problem, as articulated by Daniels (2021), is that many attitudes toward resilience suggest it is something completely accessible through individual control. The underlying assumption is that individuals learn resiliency strategies from important others (e.g., family of origin) and are expected the then enact those strategies in their own lives. This idea of a “learned resilience” is like the other side of the coin from the theory of “learned helplessness.” There is a body of research on resilience that grow in complexity with each new wave (Henry et al., 2015); however, to everyday folks in day-to-day practice, “resilience” is perceived in myriad ways with diverse definitions of what constitutes “adversity,” “overcoming,” “success,” what tradeoffs are acceptable, and where the locus of control for resilience lies Anderson and Williams, (2018); Daniels, (2021). While some research has shown that “learned” attitudes and behaviors can shape cognitive functions and stress responses (Kim et al., 2016), perceived locus of control is also a critical factor in understanding learned resilience and helplessness (Seligman, 1972). Situational circumstances beyond a person's control that exert a negative influence on their life experiences and outcomes can “significantly debilitate organisms: they produce passivity in the face of trauma, in the ability to learn that responding is effective, in emotional stress…and possibly depression” (Seligman, 2011, p. 7050).

People respond to adverse circumstances in ways that minimize what they perceive to be the worst, most harmful, or most uncomfortable consequences—and the rationale in deciding what those are is intensely personal. Someone outside of their situation and without their same individual traits or background experiences might evaluate their situation in a completely different way (stemming from a different degree of agency). Resilience has been attributed to elements of human agency, such as optimism (Elder and Hitlin, 2007; Seligman, 2011), but no known studies have examined whether someone's personal sense of agency shapes how they perceive (or blame) others for their behavior or circumstances (resilient or adverse). Agency is conceptualized as a multifaceted construct encompassing three interrelated but distinct components: self-efficacy (belief in one's capabilities), optimism (positive future expectations), and planfulness (strategic thinking about goals), following Elder and Hitlin's (2007) framework. While these components often covary, they may operate differently across contexts. For instance, parental support may foster optimism while peer relationships build self-efficacy (Schoon and Heckhausen, 2019). The current study examines whether an individual's degree of agency (framed as optimism, planfulness, and self-efficacy; Elder and Hitlin, 2007) plays a role in their fundamental attribution errors toward others. In other words, the question is whether people with a stronger sense of human agency judge others more harshly (i.e., in racist, sexist, or classist ways) than people with a lower sense of agency. Given empirical evidence that agency plays a critical role in one's own development, it is hypothesized that agency will also play an important role in blaming attitudes toward others.

More positive and compassionate attitudes toward others have been found among women than men (Eisenberg and Lennon, 1983). Particularly, adolescent girls have shown greater perspective-taking and higher and more stable levels of empathy than adolescent boys (Van de Graaff et al., 2014). The more empathetic attitudes of adolescent girls may partly be attributed to their greater closeness with mothers (Heller et al., 2006). Moreover, research suggests that fathers were much more involved with sons and tended to concentrate on more instrumental facets of support, whereas mothers tended to be more supportive across genders in the traditional, affective sense (Starrels, 1994). These differences in parental involvement may result in differing interaction styles that lead to differences in parent-child relationship quality. Furthermore, Smith et al. (2016) propose that adolescent development of socio-cognitive characteristics, such as social attitudes and agency (which have been shown to be influenced by gendered parent-child relationships Cooke et al., (2019); Nunes et al., (2022), increases perspective taking, which facilitates more favorable attitudes toward disadvantaged groups. Because girls develop social-cognitive abilities earlier than boys, girls may show greater perspective-taking and thus more positive attitudes toward disadvantaged groups than boys. Given these differences, the current study frames gender as a potentially important moderator of parent-child relationship quality, internalizing characteristics, and attitudes toward others (and how these variables relate to each other).

Racial bias in social behavior has been well-documented and can be understood from a historical perspective or interpreted in terms of sociopolitical factors (e.g., socioeconomic status). Empathetic attitudes play a key functional role in social behavior. Particularly, individuals may show different responses toward others depending on in-group and out-group biases and negative or positive contexts (Han, 2018; Neuman et al., 2013). Biological research has explained such racial differences in empathetic attitudes of members of one racial group toward other races as involving differing neurological responses (Chiao and Mathur, 2010). For example, neuroimaging research has demonstrated that racial majority group members, show greater fusiform and parahippocampal response when perceiving own-race faces, and either heightened or attenuated amygdala response to other-race faces. There are also socio-historical underpinnings for biased attitudes that can exist outside of and within racial/ethnic groups. These attitudes can range from mild prejudice to extreme xenophobia (Landi et al., 2020). Although prejudicial attitudes are known to be harmful to individuals and their social environments, they are also becoming more prevalent and vocal in polarized public sentiments (Landi et al., 2020).

**Figure 1:**
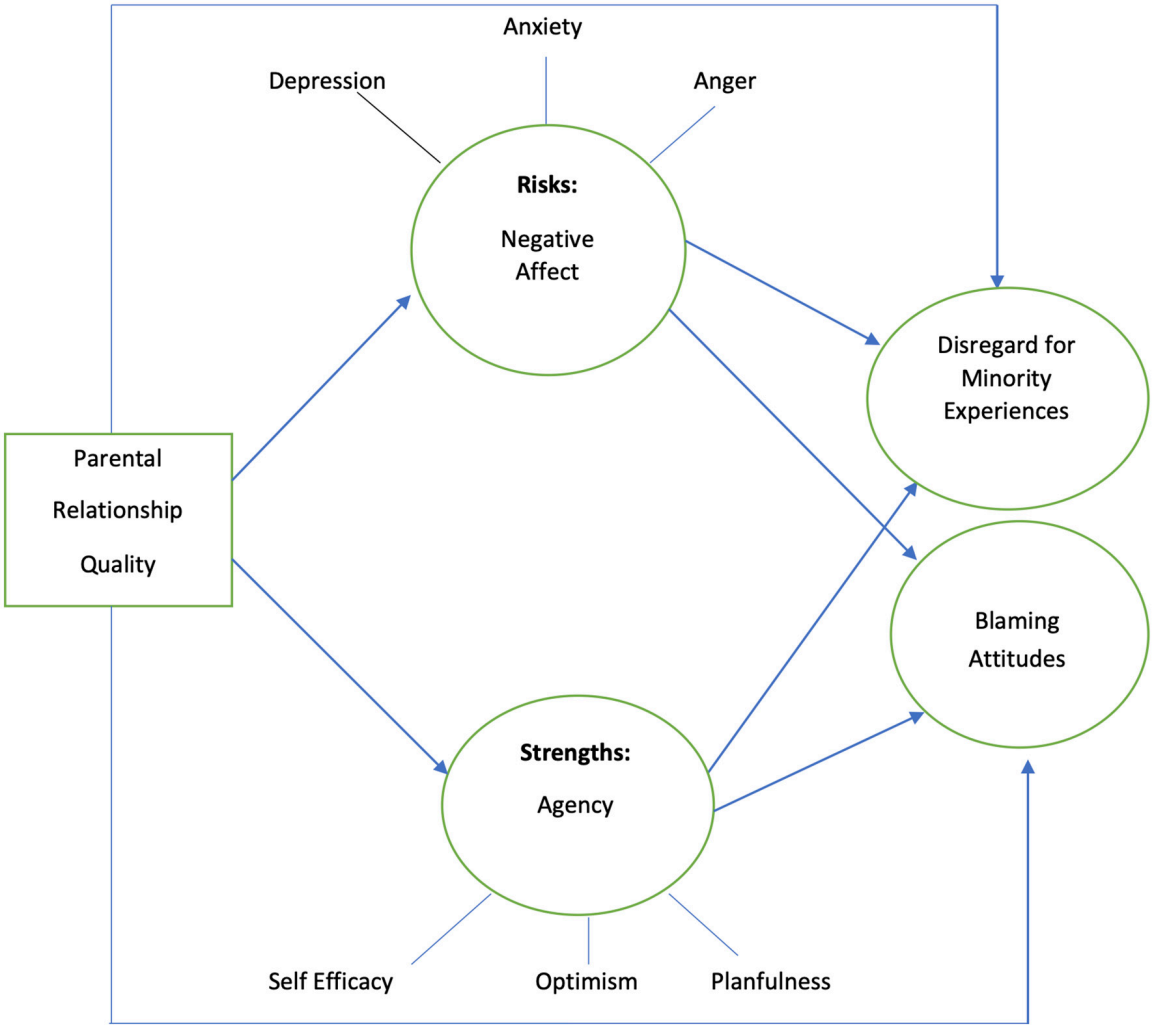
Theoretical model. Factor analysis supported three factors of Agency while PROMIS measure and the attitudes measure theorized three factors for negative affect and two factors for attitudes, respectively.

Figure 1 illustrates the theoretical model of the current study. A close relationship with parents is hypothesized to decrease conscious bias (i.e., blaming or disregarding attitudes) held by youth toward people experiencing adverse socioeconomic experiences and poverty, as well as complex minority social experiences such as sexism, racism, and heterosexism. Parental influence on adolescent beliefs and prejudicial attitudes toward others may be shaped through parental attachment, socialization, and role modeling, leading to the transfer of knowledge, understanding, and attitudes (Liable, 2007; Miklikowska, 2015). This direct effect is hypothesized to be mediated by both negative affect and agency.

Better relationships with parents are hypothesized to lead to lower negative affect while leading to higher agency (McAdams et al., 2017). Greater negative affect is hypothesized to decrease disregard for minority social experiences and blaming attitudes, while greater agency increases these forms of conscious bias, consistent with the Fundamental Attribution Error (Ross, 1977; O'Brien, 2003). Human agency, or the “capacity, willingness, and ability” of a person to actively participate in and direct their life experiences, is intricately linked with how they see themselves and others (Elder and Hitlin, 2007; Williams and Merten, 2014, p. 1,565). Seeing themselves as both competent and influential in life and having the capacity to attain an “ideal” possible self (Arnett, 2004; Bandura, 1982; Little et al., 2006; Schwartz et al., 2005) may be an important predictor of how a person views others. It may also shape whether they attribute others' challenges and successes as stemming from their own personal traits vs. situational circumstances (Ross, 1977).

## Methods

### Participants and procedure

The research team obtained IRB approval from the University of Arkansas-Fayetteville (protocol #1805121708) and participant informed consent prior to collecting a sample of 702 18-year-olds from across the United States. These data were collected using Qualtrics online panel data. Prior research indicates the validity of this data collection strategy (Walter et al., 2018; Boas et al., 2020). Participants were asked to self-report on several dimensions of their lives, including family, social, and mental health. Race/ethnicity of the sample included White (69%); Black (12%); Hispanic (8%); Asian (5%); Multi-racial (3%); and Native American (2%). A total of 80% of the sample were women.

### Measures

#### Parent relationship quality

The question asked, “I have a good relationship with my parents or childhood caregivers.” This item was assessed from 1 (Strongly Disagree) to 4 (Strongly Agree). Several studies have shown good validity and reliability of single vs. multiple item measures (Ang and Eisend, 2018). Single items have the benefits of being parsimonious in administration, participant satisfaction, efficient data processing, and minimizing ambiguity (Allen et al., 2022). Authors have previously published research using this same single time to indicate parental relationship quality or closeness (Blinded for Review). Parent closeness has been considered a valid proxy for poor and strained parent-child relationships. This item also shows good predictive validity with significant correlations with depressive symptoms (b = −0.27, *p* < 0.01), overall emotional health (b = 0.25, *p* < 0.01), overall physical health (b = 0.21, *p* < 0.01), and educational aspirations (b = 0.10, *p* < 0.05).

#### Negative affect

The 21-item PROMIS (2021) scale asked on a 5-point Likert scale from 1 (never) to 5 (always) questions, such as if in the past 7 days the adolescent felt fearful, uneasy, nervous, anxious, tense, worthless, helpless, depressed, hopeless, angry, and annoyed. These 21 items are divided into three factors: depressive, anxiety, and anger symptoms. The individual items within each factor have an acceptable internal consistency, Cronbach alphas of 0.93 (8 items, depressive symptoms), 0.96 (8 items, anxiety symptoms), and 0.93 (5 items, anger symptoms), and 0.97 for all 21. The PROMIS measures have good validity shown in numerous prior studies (e.g., DeWalt et al., 2007; Rothrock et al., 2010; Singh et al., 2021).

#### Agency

The 11-item scale (Hitlin and Elder, 2007) measured self-efficacy, optimism, and planfulness of adolescents on a Likert-type scale from 1 (strongly disagree) to 4 (strongly agree). The items asked questions such as “You have a lot of energy,” “how often during the past week did you feel hopeful about the future?”, and “when making decisions, you generally use a systematic method for judging and comparing alternatives.” Factor analysis confirmed three factors of self-efficacy, optimism, and planfulness. The internal consistency Cronbach alpha of the items pertaining to the individual factors is acceptable, with 0.54 (4 items, self-efficacy), 0.5 (3 items, optimism), and 0.74 (4 items, planfulness), and 0.71 for all 11. See Elder and Hitlin (2007) for a more robust explanation of the reliability and validity of using these items as indicators of the agency construct.

#### Conscious bias

**Figure 2:**
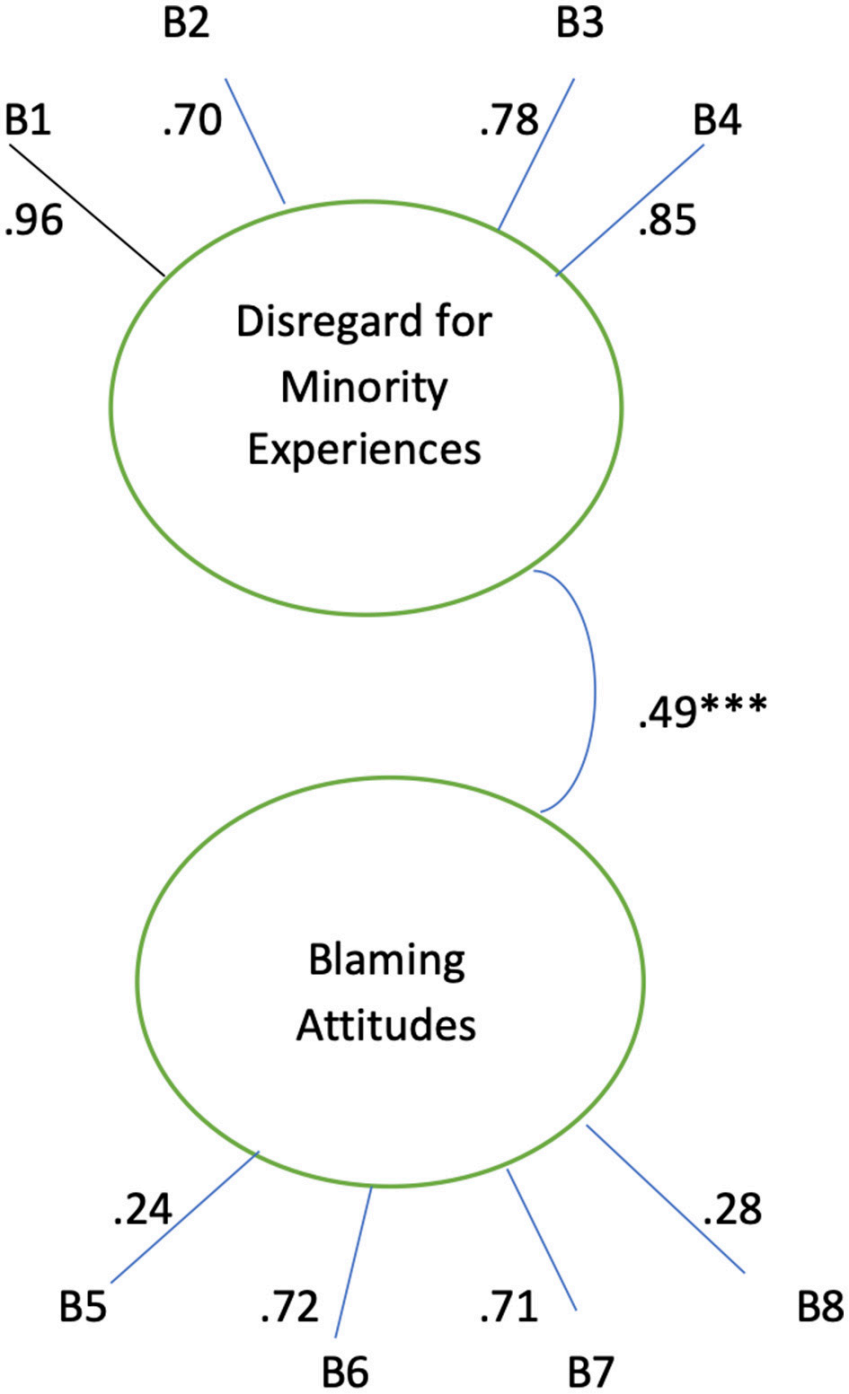
Confirmatory factor model, standardized coefficients. Model fit indices: *X*^2^/df: 4.01, RMSEA 0.08, CFI 0.96, ***p < 0.001, Significant errors correlated in the indicators. “Disregard for minority social experiences” includes racism, Sexism, heterosexism, and in favor of expressing “socially unaccepted” opinions. Standardized loadings of indicators are shown. All the loadings were statistically significant (*p* < 0.01).

Eight items were included to assess participants' conscious disregard for minority social experiences and blaming attitudes measured on a Likert-type scale from 1 (strongly disagree) to 4 (strongly agree). The items for disregard for minority social experiences asked questions such as “being lesbian, gay, bisexual, transgender, or questioning one's sexuality is a choice people make,” “racism is not a major problem in the United States', and “sexism, or unequal treatment of women compared to men, is not a major problem in the United States.” The items for blaming attitudes asked questions such as “people need to learn to help themselves,” “people become poor because of choices they have made,” and “homelessness is a choice people make because they don't want to work.” Confirmatory factor analysis affirmed the existence of these two separate dimensions of conscious bias, disregard for minority social experiences and blaming attitudes, with good discriminant validity, acceptable model fit, low to medium correlation between constructs, and indicator loadings (Figure 2). The four disregarding items had an acceptable internal consistency Cronbach alpha of 0.64, while the four blaming items also had an acceptable internal consistency Cronbach alpha of 0.54.

#### Demographic variables

To measure gender, participants were asked the following: “With which gender do you most identify?” Response options included (1 = male, 2 = female, 3 = Other; please specify). Race/Ethnicity was measured by asking participants the following: “Select the race/ethnicity with which you most closely identify?” Response options included (1 = Black, 2 = White, 3 = Hispanic, 4 = Native American, 5 = Asian, 6 = Multicultural, and 7 = Other; please specify). Income/poverty was measured through the indicator item, “How often did you feel that you didn't have enough to eat, had to wear dirty clothes, and had no one to protect you?” (one item measured on a Likert-type scale from 1 (never) to 3 (more than once). This was used as a control variable in the analysis. Income/poverty was included as a control because socioeconomic status shapes both parent-child relationships and attitudes toward disadvantaged others Kraus et al., (2012).

### Analysis

The analysis was conducted using MPlus Version 8 software (Muthén and Muthén, 2017). Preliminary analysis consisting of frequencies, descriptive statistics, and variable diagnostics was conducted. Structural Equation Modeling and Multi-Group Analysis were used to test the hypothesized associations. Bootstrap procedures were used to test the indirect effects with 2.5% above and below the estimate to create the 95% Confidence Interval cut points. Full Information Maximum Likelihood (FIML) procedure, which estimates model parameters and standard errors from all available data (Enders and Bandalos, 2001), is used in the analysis. We used the comparative fit index (CFI > 0.95) and root mean square error of approximation (RMSEA < 0.08), and the Chi-Square/df ratio to evaluate model fit (Hu and Bentler, 1999), while using the chi-square difference test for group differences in associations. The sample collected was primarily women (80%), and while sample sizes were small in group testing, the model fit was satisfactory, thus giving confidence that differences between groups were due to effects and not sample sizes. The trends should be interpreted with caution as the group sizes were different; however, as the model fit is good, the differences were acceptable. Group differences among black and white participants were examined. There were not enough participants within the other race/ethnicity groups to conduct group analyses. Missing data ranged from 2% to 8% across all variables of interest in this study. The FIML procedure was used to address missingness. The estimation assumes data are missing at random (MAR). Sensitivity analyses using multiple imputation yielded similar results.

## Results

Table 1 shows bivariate correlations among major study variables. Poverty and parent relationship quality were negatively correlated. Better relationship quality was correlated with greater self-efficacy, optimism, and planfulness. However, relationship quality was negatively correlated with depression, anxiety, and anger. Overall, three key findings emerged: (1) parent-adolescent relationship quality indirectly influences conscious bias through negative affect and agency; (2) these pathways differ by gender and race/ethnicity; and (3) agency's role in bias formation is particularly complex for minority youth and males. The following analyses support these findings.

**Table 1:** Bivariate correlations among study variables.

**Variables**	**1**	**2**	**3**	**4**	**5**	**6**	**7**	**8**	**9**
1 Poverty	–								
2 AG_SF	−0.03	–							
3 AG_Opt	−0.16^**^	0.27^**^	–						
4 AG_Plan	0.05	0.32^**^	0.32^**^	–					
5 NA_Dep	0.23^**^	−0.21^**^	−0.24^**^	−0.04	–				
6 NA_Anx	0.27^**^	−0.23^**^	−0.40^**^	−0.09^*^	0.75^**^	–			
7 NA_Ang	0.22^**^	−0.17^**^	−0.25^**^	−0.05	0.67^**^	0.67^**^	–		
8 DME	−0.02	0.23^**^	0.08	0.08	−0.17^**^	−0.16^**^	−0.08^*^	–	
9 BA	0.01	0.16^**^	0.06	−0.05	−0.21^**^	−0.14^**^	−0.11^**^	0.48^**^	–
10 PRQ	−0.23^**^	0.09^*^	0.22^**^	0.14^**^	−0.26^**^	−0.31^**^	−0.27^**^	0.07	0.03

^*^p < 0.05, ^**^p < 0.01.

AG_SF, Agency-Self Efficacy; AG_Opt, Agency-Optimism; AG_Plan, Agency-Planfulness; NA_Dep, Negative Affect-Depression; NA_Anx, Negative Affect-Anxiety; NA_Ang, Negative Affect-Anger; DME, Disregard for Minority Experiences; BA, Blaming Attitudes; PRQ, Parental Relationship Quality.

Table 2 presents descriptive statistics by gender and race/ethnicity. Parent relationship quality, blaming attitudes, disregard for minority social experiences, and negative affect were all significantly different by gender.

**Table 2:** Descriptive statistics of study variables.

**Variable**	**Minimum**	**Maximum**	**Mean**	**Std deviation**	**Variance**
Poverty	1	3	1.45	0.77	0.59
Gender	1	3	1.81	41	0.17
Parental rel. quality	1	4	3.10	0.87	0.75
Agency-self efficacy	4	16	10.52	2.21	4.89
Agency-optimism	3	12	9.46	1.72	2.96
Agency-planfulness	4	16	12.22	2.31	5.32
Negative affect—dep	8	40	23.09	8.39	70.44
Negative affect—anxiety	8	40	22.41	9.63	92.67
Negative affect—anger	5	25	15.16	5.61	31.47
Disregard minority exp	4	16	9.55	2.08	4.33
Blaming attitudes	4	16	8.78	2.67	7.15

Figure 2 displays good discriminant validity between the latent constructs of “Disregard for Minority Social Experiences” and “Blaming Attitudes.” The correlation between the two constructs is not high (0.49, *p* < 0.001), establishing good discriminant validity of the two latent constructs. Thus, the two dimensions of attitude are justified by the CFA results. The indicators of the two latent factors are within acceptable ranges. The fit indices of the confirmatory factor model are also acceptable (*X*^2^/df: 4.01, RMSEA 0.08, CFI 0.96).

**Figure 3:**
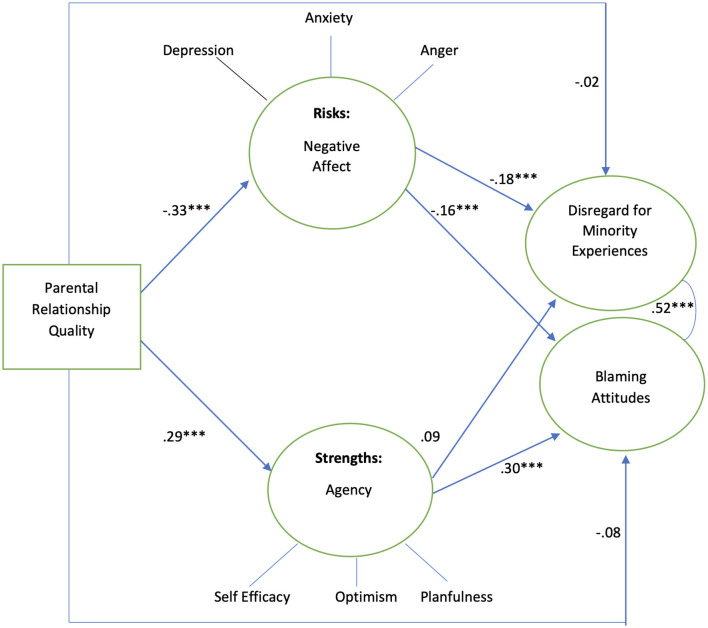
Testing full sample, standardized coefficients. Controlling for poverty, *n* = 702. Model fit indices: *X*^2^/df 3.55, RMSEA 0.06, CFI 0.90, ***p < 0.001. *R*^2^ Disregard for minority experiences 4.2%, *R*^2^ blaming attitudes 10.8%.

Figure 3 shows the results for analyzing the model using the total sample which shows significant mediational associations between parent relationship quality and attitudes of adolescents. The results for analyzing the model using the total sample showed significant mediational associations between parent relationship quality and attitudes of adolescents. Better relationships with parents were negatively associated with negative affect (β −0.33, *p* < 0.001) and positively associated with agency (0.29, *p* < 0.001). Greater negative affect was negatively associated with both disregard for minority social experiences and blaming (β−0.18, *p* < 0.001 and β −0.16, *p* < 0.001, respectively). Greater agency was positively associated with blaming (β 0.30, *p* < 0.01) but did not associate with disregard for minority social experiences. Parent relationship quality did not directly associate with adolescent attitudes. The model explained 4.2% of the variance in disregard for minority social experiences while explaining 10.8% of the variance in blaming.

Table 3 shows the indirect effects in the full sample model, including the estimate and the 95% confidence interval. All indirect effect paths were statistically significant.

**Table 3:** Indirect effects (standardized coefficients) in the full sample model.

**Indirect effects**	**Estimate**	**95% confidence interval**	
		**Lower limit**	**Upper limit**
PRQ-NA-DME	0.06^**^	0.02	0.11
PRQ-NA-BA	0.05^*^	0.01	0.10
PRQ-AG-BA	0.09^*^	0.04	0.19

PRQ, Parental Relationship Quality; NA, Negative Affect; AG, Agency; DME, Disregard for Minority Experiences.

^*^p < 0.05, ^**^p < 0.01.

**Figure 4:**
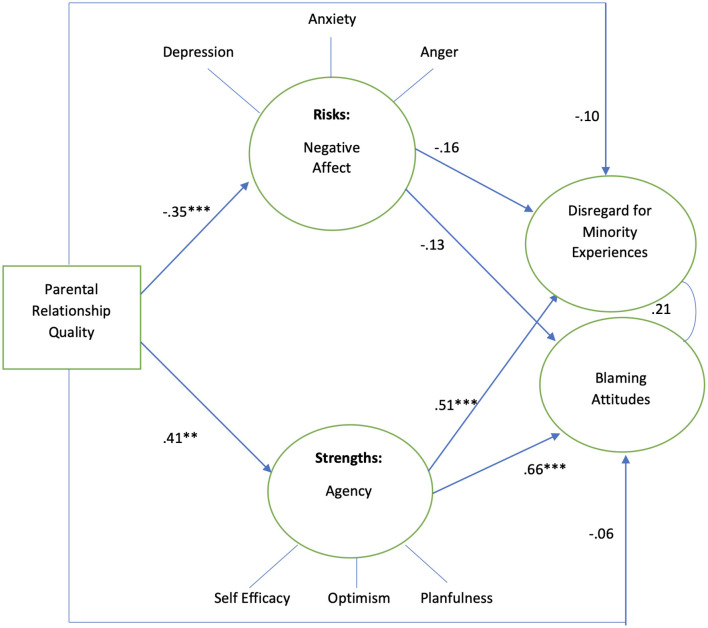
Males sample, standardized coefficients. Controlling for poverty, *n* = 115. Model fit indices: *X*^2^/df 2.09, RMSEA 0.09, CFI 0.82, **p < 0.01, ***p < 0.001. *R*^2^ Disregard for minority experiences 26.3%, *R*^2^ blaming attitudes 45.8%.

Figure 4 shows model results for male adolescents, which indicated mediation by negative affect and partial mediation by agency in the association between parent relationship quality and adolescents' attitudes. Among male adolescents, the results showed the mediation by negative affect and partial mediation by agency in the association between parent relationship quality and adolescents' attitudes. Better parent relationship is positively associated with agency, while it is negatively associated with negative affect (β 0.41, *p* < 0.01, β −0.35, *p* < 0.001, respectively). Greater negative affect was negatively associated with both disregard for minority social experiences and blaming attitudes (β −0.16, *p* < 0.05 and β −0.13, *p* < 0.05, respectively). Greater agency was positively associated with disregard for minority social experiences and blaming (β 0.51, *p* < 0.05 and β 0.66, *p* < 0.05, respectively). The model explained 26.3% of the variance in disregard for minority social experiences while explaining 45.8% of the variance in blaming.

**Figure 5:**
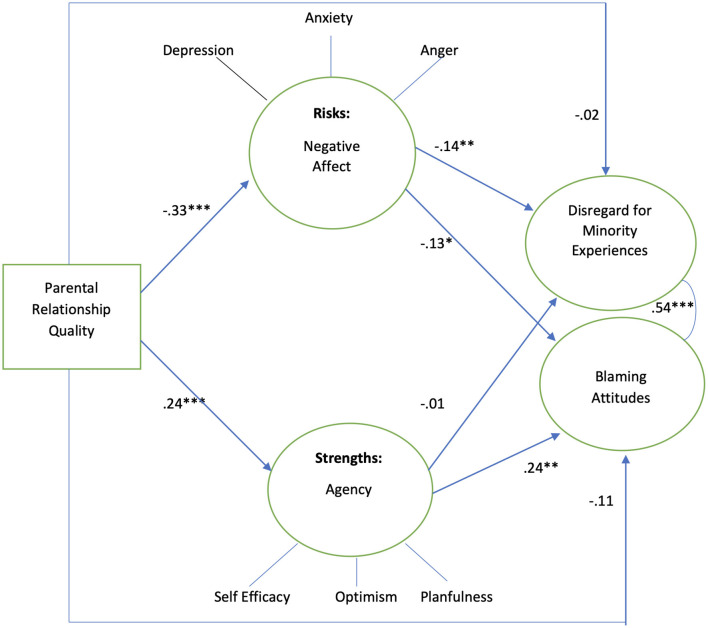
Females sample, standardized coefficients. Controlling for poverty, *n* = 487. Model fit indices: *X*^2^/df 2.91, RMSEA 0.06, CFI 0.91, *p < 0.05, **p < 0.01, ***p < 0.001. *R*^2^ disregard for minority experiences 2.4%, *R*^2^ blaming attitudes 7%.

Among female adolescents, the results also showed mediation by negative affect and partial mediation by agency in the association between parent relationship quality and adolescents' attitudes (Figure 5). Parent relationship quality is negatively associated with negative affect, while it is positively associated with agency (β −0.33, *p* < 0.001 and β 0.24, *p* < 0.001, respectively). Greater negative affect was negatively associated with both disregard for minority social experiences and blaming (β −0.14, *p* < 0.001 and β −0.13, *p* < 0.05, respectively). Greater agency was positively associated with blaming but did not associate with disregard for minority social experiences (β 0.24, *p* < 0.05 and β −0.01, *p* > 0.05, respectively)—the opposite results as those for male adolescents in the association between agency and disregard for minority social experiences. The model explained 2.4% of the variance in minority social experiences, while it explained 7% of the variance in blaming.

**Figure 6:**
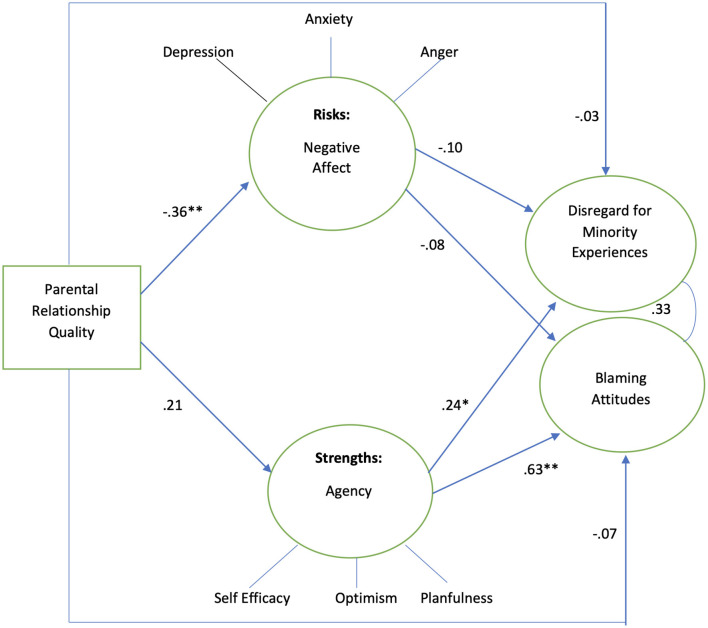
Black sample, standardized coefficients. Controlling for poverty, *n* = 70. Model fit indices: *X*^2^/df 1.17, RMSEA 0.05, CFI 0.94, *p < 0.05, **p < 0.01. *R*^2^ disregard for minority experiences 7.4%, *R*^2^ blaming attitudes 39.6%.

Results for the black sample showed no mediation by negative affect and agency on the association between parent relationship quality and adolescents' attitudes (Figure 6). Relationship quality was negatively associated with negative affect but did not associate with agency (β −0.36, *p* < 0.01 and β 0.21, *p* > 0.05, respectively). In turn, greater negative affect was not associated with either disregard for minority social experiences or blaming. However, greater agency was positively associated with blaming (β 0 .63, *p* < 0.01). The model explained 7.4% of the variance in disregard for minority social experiences and 39.6% of the variance in blaming.

**Figure 7:**
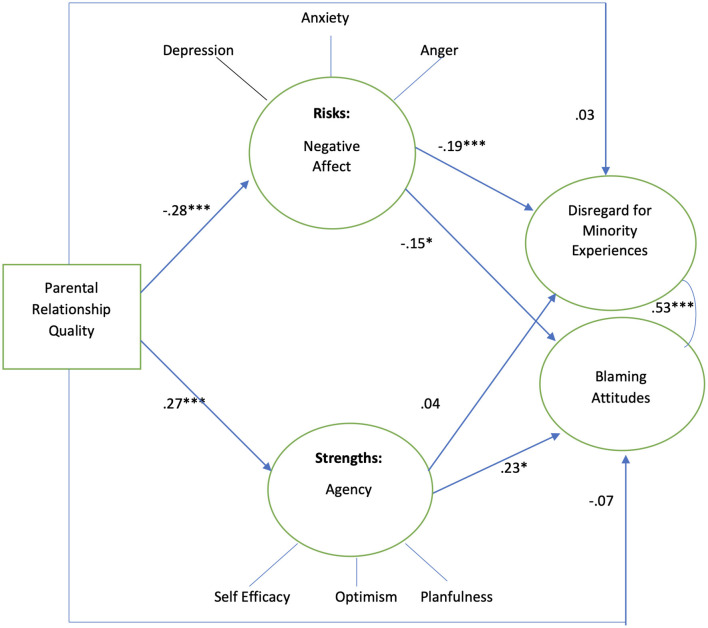
White sample, standardized coefficients. Controlling for poverty, *n* = 429. Model fit indices: *X*^2^/df 2.82, RMSEA 0.07, CFI 0.90, *p < 0.05, ***p < 0.001. *R*^2^ disregard for minority experiences 4.6%, *R*^2^ blaming attitudes 7.3%.

Results using the white adolescent sample showed mediation by negative affect in the association between parent relationship quality and disregard for minority social experiences and blaming (Figure 7). Relationship quality is negatively associated with negative affect (β −0.28, *p* < 0.001). Greater negative affect was negatively associated with disregard for minority social experiences and blaming (β −0.19, *p* < 0.001 and β −0.15, *p* < 0.01, respectively). While parent relationship quality was positively associated with agency (β 0.27, *p* < 0.01), greater agency in turn was positively associated with blaming (β 0.23^*^, *p* < 0.05). While for white adolescents the association between negative affect and adolescent attitudes was significant, for black adolescents these associations were not significant. The model explained 4.6% of the variance in disregard for minority social experiences, while it explained 7.3% of the variance in blaming.

Multi group testing using MPlus revealed that the association between Disregard for Minority Social Experiences and Agency was significantly different between genders (males β 0.51, *p* < 0.001, females β −0.01, ns), and the association between Disregard for Minority Social Experiences and Agency was also significantly different between white and black groups (white β 0.04, ns, black β 0.24, *p* < 0.05). These results indicate the association between Disregard for Minority Social Experiences and Agency was significantly positive and stronger for male adolescents and black adolescents. Comparisons for Hispanic, Asian, Native American, and multiracial groups were not conducted due to insufficient sample sizes (n < 60 per group), preventing reliable parameter estimation.

## Discussion

Results provide support for the theorized model in that parent-adolescent relationships can be linked with disregard for or negativity toward others' experiences through adolescent mental wellness and sense of agency. Findings indicate that disregard for minority experiences and blaming attitudes toward victims of adversity are related and often co-occurring behaviors. However, these pathways were nuanced by gender and race/ethnicity. Specifically, while parental relationship quality was important for all youth in the sample, the mechanisms linking relationship quality with apathetic or malevolent attitudes toward minorities and disadvantaged others varied by gender and race/ethnicity.

Consistent with previous research, when youth feel close to their parents (a sentiment encompassing a wide variety of parenting dynamics (Mak et al., 2020), they report better mental health and greater human agency. Parental relationship quality and mental health were universally linked for all youth in the sample, and, when analyzed by gender, were important for both adolescent boys and girls (with a greater magnitude for boys in terms of agency). However, while parent-youth relationship quality and agency were significantly linked among white youth, this association was not found for black participants. Previous research has suggested agency may not be present, may be experienced the same among diverse individuals, and that the scope of what constitutes capacity for function and resilience across racial/ethnic groups needs expansion (Williams and Merten, 2014). This may also be true in terms of the parenting environment and how racially/ethnically diverse families foster agency among their sons vs. their daughters. However, parenting and agency (assessed as planfulness, optimism, and efficacy) were significantly linked for male and female teens in the present study. These findings add to developmental theory in several ways. First, they extend Kohlberg's (1987) moral development framework by showing how parent-adolescent attachment shapes moral reasoning about disadvantaged others. Second, they support social domain theory (Turiel, 2002) by demonstrating how personal relationships influence judgments in the moral domain. Third, they align with social cognitive domain theory (Smetana, 2006), suggesting parental relationships provide the scaffolding for developing complex social judgments about inequality.

When examining the pathways linking negative affect (depressive, anger, and anxiety symptomology) and agency with disregard for minority experiences and blaming attitudes, an interesting picture developed. Surprisingly, greater negative affect was associated with lower conscious bias (less disregard and blaming). This counterintuitive finding may reflect heightened empathy among those experiencing their own struggles, consistent with the wounded healer hypothesis (Zerubavel and Wright, 2012). It is likely that individuals experiencing higher depressive symptoms, anger, and anxiety tend to feel more apathetic to the plight of others. This could be due to misinterpreting or downplaying the struggles of others (Janezic and Arsenault, 2021) or not having the mental capacity for empathetic thought because of their own internalizing problems (Önal et al., 2021). It is well-established that individuals experiencing internalizing problems (particularly depression) have diminished motivation (Smith, 2013). Lockwood et al. (2017) found empathic attitudes and behaviors require a degree of motivation and intentionality (vs. being an automatic perspective or action), which adds additional rationale for participants' seemingly apathetic attitude toward the adverse experiences of minorities and disadvantaged individuals. However, negative affect was unrelated to disregard or malevolent views among male and black youth. It is likely that other contextual factors (such as socialization processes, personal experiences, and awareness of interpersonal and systemic racism and discrimination) are more relevant to (or intercorrelated with) these views than solely individual mental health. On average, male and black participants had higher conscious bias values for blaming attitudes and disregard for minority experiences (compared to female and white participants). While these patterns in attitudes appear similar, they are likely due to different socialization processes where one group (males) is socialized to be agentic as traditionally defined, and the other is socialized to overcome historic adversities and may then hold their peers to more critical standards. Understanding these similarities and differences is a future research direction that is important for understanding the nuances of human agency from intersectional perspectives and how it interplays with biased or empathetic attitudes.

Interestingly, while human agency only explained differences in blaming attitudes in the full sample, there were notable differences in this mechanism by gender and race/ethnicity. First, the link between agency and disregard was only significant for men, while agency was related to blaming attitudes for both men and women. A similar pattern was found based on race/ethnicity, where agency and disregard were only significantly linked for black youth, while agency was important for all groups in terms of blaming attitudes. While a link between human agency and empathic perspective-taking makes sense due to the nature of agency (and emphasis on locus of control), it is possible that this is even more pronounced for minority youth. Given a stream of research pointing to structural, instrumental, and psychosocial challenges experienced by minority youth, families, and communities (heavily shaped by systemically discriminatory beliefs and practices (e.g., Gaylord-Harden et al., 2018; Liu et al., 2017; Wickrama et al., 2012), those who have learned resilience instead of helplessness may hold other minorities or disadvantaged people to a higher standard than individuals without culturally victimized histories. In other words, because of historical and evolving experiences of interpersonal and systemic prejudice and discrimination, minority youth may place even more emphasis on agency as a promotive factor for resiliency. It is interesting that the same pattern was found for men, but for potentially very different reasons. While minority youth may be embedded in a historically marginalized context that makes resilient youth more critical of others, men have been described as thinking more instrumentally (vs. affectively, Hoffman, 1977) and are typically socialized to be more dominant (Milfont and Sibley, 2016)—traits and socialization processes that can also make them critical of others (especially those experiencing hardship who may appear weak. Gendered socialization is found in families across racial/ethnic groups, and future studies of victim blaming or racial malevolence should include a more intersectional focus on young people's socialization by culture.

Taken together, findings point to key areas for development in parent-child relationships that focus on diversity and equity as critical elements of empathy training. One of the leading resources for family scientists and practitioners is the National Council on Family Relations, which certifies parent educators through its Family Life Education credential. National Council on Family Relations (2021) provides a host of resources for talking to children about racial injustice and tragedies; however, there appear to be many opportunities to enhance resources on parenting youth who are coming of age in a digital era, amidst racial and income-driven discrimination and violence. All youth would likely benefit from learning how to take the perspectives of others, especially others who are experiencing marginalization or economic adversity. Given that parent-adolescent relationship quality indirectly reduces bias through psychological mechanisms, interventions should focus on strengthening these bonds rather than solely providing educational resources. Programs enhancing parent-child attachment security, such as *Attachment-Based Family Therapy* (Ewing et al., 2015), may be more effective than didactic anti-bias training alone. For clinical practice, findings suggest interventions like *Tuning in to Teens* (Havighurst et al., 2009; Kehoe et al., 2014) that enhance parent-child emotional connection may indirectly reduce youth bias. Educational interventions should incorporate parent components, as programs like the *Intergroup Contact Theory*-based interventions show greater effects when parents are involved (Hughes et al., 2016).

Results from the present study show that parent-child relationships are important in shaping youth malevolence toward disadvantaged others—even among youth who may have overcome the very disadvantages they are now criticizing others for. The study limitations include using cross-sectional data where a longitudinal design would have strengthened the causal arguments, the associational nature of results questioning the directions of association, and limiting the impact on conclusions and their implications. The single-item parent relationship quality variable measured perceived relationship quality rather than emotional closeness specifically. Following Laursen and Collins (2009), relationship quality encompasses multiple dimensions, including closeness, support, and conflict absence. Although single-item measures have demonstrated validity in specific contexts (e.g., Bergkvist and Rossiter, 2007), they still pose limitations. The parent relationship quality item showed predictive validity through correlations with mental health outcomes; however, future research should employ multi-item/multidimensional scales for more robust measurement. The lower internal consistency of some agency subscales in this study may attenuate observed relationships and increase Type II error risk (Schmitt, 1996). These reliability issues may explain why the agency showed inconsistent effects across groups. Moreover, the sample size limitations in gender balance and the limited sample of race/ethnic minorities prevented further group analyses. The sample restriction to 18-year-olds limits developmental insights, as bias formation occurs across adolescence (Raabe and Beelmann, 2011). The gender imbalance (80% female) and racial composition (69% white) restrict generalizability, particularly for understanding minority male experiences. Future research should employ stratified sampling to ensure adequate representation across demographic groups. Having a multiple respondent design would have strengthened the reliability of the data, avoiding reporter bias. Findings warrant additional, more robust, and longitudinal studies of parent-child relationships, as they relate to shaping empathic attitudes. Future research might also examine potential benefits of infusing diversity education and activism into general parent education, vs. continually outsourcing that knowledge development to non-family contexts or limiting the scope to advocacy among families with vulnerable children. A longitudinal approach in future research on this topic would also allow for tracking of the influence of parents on shaping biased attitudes across adolescence and into early adulthood, which would account for the shifting influences of family vs. peers.

## Conclusion

The current study examined the role of parent-adolescent relationship quality in shaping views toward minorities and individuals experiencing economic adversity. Results suggest that these close bonds are important for shaping biased attitudes, and potentially empathy, through better psychosocial health. However, while affect was universally important for blaming attitudes among study participants, agency played a more nuanced role based on gender and race/ethnicity. There is important work needed to further expand the conceptualization of human agency that is sensitive to gender differences and gendered socialization practices, as well as potentially nuanced definitions and meanings of agency among different racial/ethnic groups and cultures. There is also a significant opportunity and need to sensitize general parent education to issues of diversity, equity, and inclusion in terms of how it shapes youth attitudes and interactions with others.

## Data Availability

The raw data supporting the conclusions of this article will be made available by the authors, without undue reservation.
